# The Association between Parent Diet Quality and Child Dietary Patterns in Nine- to Eleven-Year-Old Children from Dunedin, New Zealand

**DOI:** 10.3390/nu9050483

**Published:** 2017-05-11

**Authors:** Brittany Davison, Pouya Saeedi, Katherine Black, Harriet Harrex, Jillian Haszard, Kim Meredith-Jones, Robin Quigg, Sheila Skeaff, Lee Stoner, Jyh Eiin Wong, Paula Skidmore

**Affiliations:** 1 Department of Human Nutrition, University of Otago, Dunedin 9054, New Zealand; brittany_davison@hotmail.com (B.D.); pouya.saeedi@otago.ac.nz (P.S.); katherine.black@otago.ac.nz (K.B.);harriet.harrex@otago.ac.nz (H.H.); jillian.haszard@otago.ac.nz (J.H.); sheila.skeaff@otago.ac.nz (S.S.); 2 Department of Medicine, University of Otago, Dunedin 9054, New Zealand; kim.meredith-jones@otago.ac.nz; 3 Cancer Society Social and Behavioural Research Unit, Department of Preventive and Social Medicine, Dunedin School of Medicine, University of Otago, Dunedin 9054, New Zealand; robin.quigg@otago.ac.nz; 4 Department of Exercise and Sports Science, University of North Carolina, Chapel Hill, NC 27519, USA; stonerl@email.unc.edu; 5 School of Healthcare Sciences, Faculty of Health Sciences, Universiti Kebangsaan Malaysia, Kuala Lumpur 50300, Malaysia; wjeiin@ukm.edu.my

**Keywords:** children, parents, diet quality, dietary patterns

## Abstract

Previous research investigating the relationship between parents’ and children’s diets has focused on single foods or nutrients, and not on global diet, which may be more important for good health. The aim of the study was to investigate the relationship between parental diet quality and child dietary patterns. A cross-sectional survey was conducted in 17 primary schools in Dunedin, New Zealand. Information on food consumption and related factors in children and their primary caregiver/parent were collected. Principal component analysis (PCA) was used to investigate dietary patterns in children and diet quality index (DQI) scores were calculated in parents. Relationships between parental DQI and child dietary patterns were examined in 401 child-parent pairs using mixed regression models. PCA generated two patterns; ‘Fruit and Vegetables’ and ‘Snacks’. A one unit higher parental DQI score was associated with a 0.03SD (CI: 0.02, 0.04) lower child ‘Snacks’ score. There was no significant relationship between ‘Fruit and Vegetables’ score and parental diet quality. Higher parental diet quality was associated with a lower dietary pattern score in children that was characterised by a lower consumption frequency of confectionery, chocolate, cakes, biscuits and savoury snacks. These results highlight the importance of parental modelling, in terms of their dietary choices, on the diet of children.

## 1. Introduction

Good dietary habits need to be developed during childhood, not only to improve short-term health and but also to avoid carrying unhealthy habits into adulthood, which is also associated with negative health outcomes in long term, such as increased risk of cardiovascular disease [1,2]. One key area associated with children’s dietary intake is the influence of parental diet [3,4,5]. Previous research in this area initially focused heavily on the relationships between parental and child consumption of fruit and vegetables [6]. More recent studies have looked at different food groups and/or nutrients, including a review that showed associations between child and parent intake of energy and total fat [7,8,9]. However, it is unrealistic to assume that foods are eaten in isolation. Instead, it is important to recognise that people consume meals and that there are synergistic relationships between food and nutrients [10]. To consider the diet as a whole, dietary patterns can be used. These take into account the combinations of foods consumed and have been increasingly used alongside individual dietary intake data. Dietary patterns can be derived theoretically or empirically [11]. 

Theoretical dietary patterns are used to determine how closely people adhere to a diet. For example, the Healthy Eating Index (HEI) is used to measure how well an individual’s diet conforms to the US Healthy Food Pyramid [12]. Empirically derived dietary patterns use statistical techniques, such as principal component analysis (PCA), to derive data driven patterns specific to the population of interest [11]. There is little research investigating the relationship between parent and child dietary patterns, particularly with regards to empirically derived dietary patterns. Only three studies have investigated the association between parent and child diet quality, all of which used theoretical methods to determine dietary patterns [7,13,14]. All found positive relationships between parent and child diet quality. 

The limited current literature provides an indication that dietary quality is associated between parents and children, although theoretically derived patterns based on the national nutrition guidelines differ between countries. Consequently, any significant relationships found using a particular country specific index may not be applicable to other populations. Secondly, there is a lack of research investigating the relationship between parent and child diets using empirically derived dietary patterns. The objective of the current study was to determine whether a higher parent DQI score based on the New Zealand Food and Nutrition Guidelines is associated with more healthful dietary patterns in New Zealand children aged 9–11.

## 2. Materials and Methods

### 2.1. Study Design and Participants

The present study analysed data collected as part of the Physical activity, Exercise, Diet And Lifestyle Study (PEDALS), which was a cross-sectional survey conducted in Dunedin, New Zealand between April and December 2015. Thirty out of 55 primary schools in the greater Dunedin area were invited to participate. The remaining schools were not invited as they had less than 15 Year 5 and 6 pupils on the school roll. In New Zealand, Year 5 and 6 students are typically between 9–11 years old. School principals were sent study invitation packs and if they agreed to participate, the research team visited the school to present at a Year 5 and 6 assembly. Eligible students were given packs to take home, containing letters of information and consent forms for themselves and their parents. Written parental consent and written child assent were both required in order for both the child and parent to participate. The study was conducted in accordance with the Declaration of Helsinki, and all procedures involving human subjects were approved by the University of Otago Human Ethics Committee

### 2.2. Data Collection

Participating children completed two questionnaires during class time, with assistance for reading questions given from the research team when necessary. The first questionnaire contained questions about the child (date of birth, age, sex, and year at school), their food and drink consumption, and known correlates of these. The second questionnaire focused on physical activity and its correlates. Trained research assistants also measured children’s height, weight, waist circumference, handgrip strength, body composition, blood pressure and arterial stiffness. Cardiovascular fitness was assessed using the 20 metre shuttle run test. Child participants also received accelerometers to wear for seven days. The children were given two further questionnaires and an accelerometer to take home to their primary caregiver/parent/guardian. The parent questionnaire covered similar topics to the children’s questionnaires. Questions on ethnicity, socio-economic status (SES), height, weight, education and health behaviours were also included. SES was assessed using the New Zealand Deprivation Index Score (NZDep13), which combines nine variables from the 2013 census, reflecting eight dimensions of deprivation, including owning a house and access to a car [15]. The deprivation index is an ordinal scale ranging from one (least deprived) to ten (most deprived). School decile is determined by the deprivation level, as measured by NZDep13, of students attending the school, with the lowest decile rating reflecting the 10% of schools nationwide with students mostly from high deprivation areas. School decile was divided into ‘Low’ (Deciles 1 to 4) ‘Middle’ (Deciles 5 to 8) and ‘High’ (Deciles 9 and 10).

The PEDALS Food Frequency Questionnaire (FFQ) was used to assess the children’s usual dietary intake. This was a 28-item non-quantitative FFQ, which incorporates questions from the Health Behaviour in School-Age Children Questionnaire [16]. The PEDALS FFQ has been shown to have acceptable relative validity and reproducibility in this age group [16]. The food items included were fruits, vegetables, milk (standard (full fat), light/semi-skimmed (contains around 1.5 g fat per 100 mL) and trim/skimmed (contains around 0.1 g fat per 100 mL), cheese, yoghurt, ice cream, processed meats, other meats, fish, fruit juice, fizzy drinks (diet and standard), breakfast cereals, bread (white and brown/wholemeal), rice, pasta, potato, potato chips, hot chips, biscuits, bakery food, snack bars, lollies/sweets, chocolate, tomato sauce/ketchup and sandwich spreads. Participants reported their usual intake from seven categories ranging from ‘Never’ to ‘Every day, more than once.’

Included in the second parent questionnaire was a dietary habits questionnaire (DHQ), which was used to assess parental dietary intake and eating habits, and was used in the 2008–2009 Adult Nutrition Survey in New Zealand [17]. This DHQ focused on food choices made over the previous four weeks. Nineteen questions were included, beginning with ten questions assessing intake of red and processed meat, chicken, fish and shellfish, hot chips, soft drinks and energy drinks, fruit juice and confectionery. Participants reported consumption from one of six categories: never; less than once a week; one to two times per week; three to four times a week; five to six times a week; seven or more times per week. There were five questions on dietary practices, such as removing fat from meat and chicken, adding salt to food, and choosing low fat and salt varieties over standard varieties. Lastly, there were four questions on type of milk, butter or margarine, bread and cooking fat used most often. Information was also collected on the highest level of education obtained by the parent participant and answers were collapsed into three groups—Secondary education until around minimum school leaving age (15 to 16 years) or equivalent, further secondary school completion (usually around 18 years), and post-secondary education. 

Missing data were imputed for certain questions from the questionnaires. For responses to be imputed, at least 75% of each set of questions, where a question had at least four sub-questions, needed to have been completed and ‘worst-case scenario’ responses were entered. For these analyses, the only data that were imputed were for the FFQ and the DHQ. The FFQ was considered a question, and each item within it a sub-question. Thirty-five data points (0.25% of the total) were imputed for the entire FFQ dataset. The DHQ was also considered a question, and each item within it a sub-question. Seventy-two data points (0.85% of the total) were imputed for the entire DHQ dataset. 

### 2.3. Statistical Analysis

Participants (child and parent pair) were excluded if they did not have complete information on all variables of interest for this analysis. For descriptive analyses, ethnicity was categorised into three groups, as in previous New Zealand surveys [17]: ‘Māori’; ’Pacific People’; and ‘New Zealand European and Other’ (NZEO) were prioritised in that order. NZEO includes those who identify as New Zealand European, as well as other groups that were too small for individual analysis, such as Indian and Korean. Due to the small number of Māori and Pacific Island participants in the sample, ethnicity was further condensed to two groups: ‘Māori and Pacific People’ and ‘NZEO’ when included in multivariate analysis. Child body mass index (BMI) was calculated using measured height and weight, *z*-scores calculated using World Health Organisation (WHO) growth charts and categorised using the WHO categorization [18]. Self-reported height and weight were used to calculate parent BMI. A BMI < 25 was categorized as healthy, 25–29.9 was overweight, and ≥ 30 was obese. Twenty-eight food items from the PEDALS FFQ were aggregated into 21 groups based on similarity in nutritional profile. Children’s dietary pattern scores were derived using PCA with varimax orthogonal rotation. Determining the number of components (patterns) was based on eigenvalues >1 and identification of the elbow in the scree plot [19]. Once the patterns had been identified, food groups within these patterns with factor loadings ≥0.2 were considered significant when naming the patterns. Skew was removed from the distribution of the scores and then scores were standardized. A dietary quality index (DQI) for parents was calculated from the DHQ data. This was slightly modified from that developed by Wong et al. for New Zealand adolescents aged 16–18 years [20]. The only modifications made to the adult index, compared to the published adolescent index, were the exclusion of the items assessing frequency of consumption of confectionery, fruit juice, processed meat, and type of cooking fat (which loaded very lowly on the index), and the inclusion of two items assessing frequency of adding salt and consumption of reduced-salt foods. This adult DQI showed good validity in the 3993 adult participants from the 2008–2009 New Zealand Adult Nutrition Survey (unpublished data). A 5-point scoring system was used, with scores ranging from 0 to 4. A response that matched a more positive dietary habit was assigned a higher score. The total diet quality score was a summation of scores from 15 items and ranged from 0 to 60. 

Mixed regression models were used to investigate associations, with school as a random effect. These were used to determine the difference between boys’ and girls’ dietary pattern scores and male and female parent DQI scores. Both unadjusted and adjusted models were run for the association between parent DQI score and child dietary patterns. Adjustment was made for parent age, sex and BMI; child age, sex, ethnicity and BMI *z*-score; and level of deprivation (NZDep13). We also ran an additional model that included parental education, but as the addition of this variable made no difference to any results this is not shown. 

Interaction terms between the DQI score and (a) who completed the DQI (mother or father) and (b) sex of the child were included to determine whether associations between dietary patterns and DQI were moderated by the sex of the parent or the child. If an interaction term was significant, then stratified results were also determined. If not significant, then the interaction term was removed from the model. Regression coefficients (95% CI) and *P* values are presented. Two-sided *p* values of < 0.05 were considered statistically significant. All statistical analysis used Stata 14.1 (StataCorp, College Station, TX, USA).

## 3. Results

**Figure 1:**
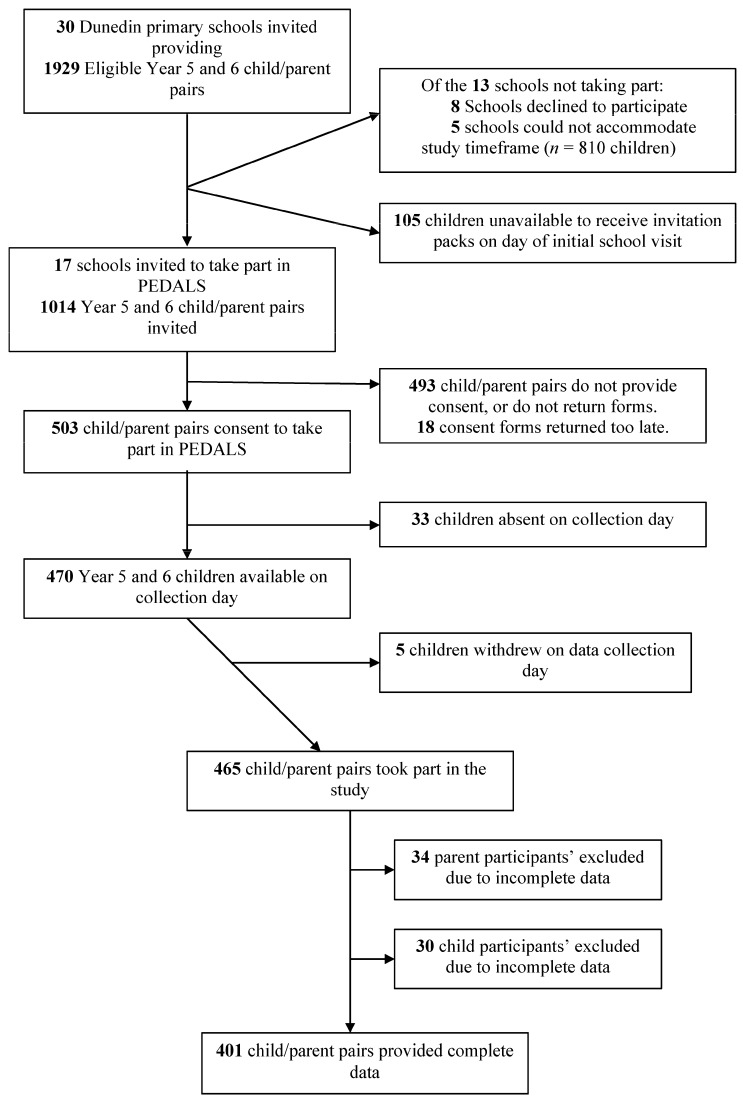
Recruitment flowchart for PEDALS.

Overall, complete demographic, questionnaire and anthropometric data was obtained from 401 child–parent pairs. Figure 1 provides an overview of school, student and parent recruitment. Seventeen of 30 invited schools took part, with 470 students available on data collection days. Of these, 468 students took part in PEDALS. The majority of students (57%) attended high decile schools (8–10) (Table 1). The mean age of the child participants was 10.2 years. The majority of child participants were of NZEO ethnicity, with 9% identifying as Māori and 3% as Pacific People. Based on the WHO BMI categories, 16% of child participants were overweight and 11% were obese. Parent participants were on average 41.6 years old and the majority of those completing the parental questionnaires were female (83.5%). Overall, 50% of parents were overweight or obese (70% of fathers and 46% of mothers). Forty-seven percent of fathers and 44% of mothers were in the lowest NZDep13 categories (1–3).

Fruits and vegetables were consumed at least once every day by 66% and 56% of children, respectively. Four percent of children consumed fizzy drinks, 4% consumed diet fizzy drinks, 5% consumed lollies/sweets and 5% consumed chocolate every day. Three percent of parents consumed fizzy drink and 5% consumed confectionery every day. Only 1% or less consumed red meat, hot chips or processed meat every day. Standard milk was the most commonly consumed milk by parents (42%) and light grain bread was the most commonly consumed bread (48%).

PCA produced two dietary patterns that explained 37% of the total variance. The patterns were a ‘Snacks’ pattern and a ‘Fruit and Vegetables’ pattern (Table 2). The ‘Snacks’ pattern loaded positively for ice cream, non-dairy drinks, white bread, pasta and noodles, salty snacks, sweet baked items, lollies/sweets, sweet snacks and sandwich spreads. The ‘Fruit and Vegetables’ pattern loaded positively for fruits, vegetables, trim milk, standard milk, cheese, yoghurt, processed meats, other meats, breakfast cereals and brown/wholemeal bread.

Boys had a significantly higher ‘Snacks’ pattern score compared to girls 0.13 (SD = 1.0) compared to −0.12 (SD = 1.0), *p* = 0.005. There was no significant difference between boys and girls for ‘Fruit and Vegetables’ pattern scores (*p* = 0.384). The mean parent DQI score was 43 (SD = 7), with a possible highest score of 60. The mean score for mothers was 44 (SD = 7), four points higher than the mean father score of 40 (SD = 6) (*p* < 0.001). After adjustment for confounders there was a significant inverse relationship between the ‘Snacks’ pattern score and parent DQI scores, with a one unit increase in parental diet quality score associated with a 0.03SD (CI: 0.02, 0.04) decrease in the child ‘Snacks’ pattern score (Table 3). There was no significant relationship between the ‘Fruit and Vegetables’ pattern and DQI score. None of the included interaction terms included in the models were significant (data not shown). 

## 4. Discussion

This is the first study globally to investigate the relationship between parental and child dietary patterns using theoretical and empirical methods of derivation, respectively. The results indicate that there is an inverse association between parent diet quality and the child ‘Snacks’ pattern.

In this study, 66% of children consumed fruit at least once daily, whereas only 4% had fizzy drinks every day, suggesting that this sample of children had relatively healthy diets. However, it cannot be concluded whether or not the children were reaching the recommended three servings of vegetables and two servings of fruit per day as this information cannot be derived from a non-quantitative FFQ. Data from a nationally representative New Zealand study conducted in 2008–2009 shows that only 30% of 5–9 years old and less than 38% of 10–14 years old were achieving both recommendations [21]. As the sample from the current study had relatively low levels of deprivation and the children were mainly from the NZEO group, it is possible that a higher proportion of children were having five servings of fruit and vegetables per day compared to the national average. However, this is purely speculation, given the nature of the FFQ used in this study.

Two dietary patterns were derived from the child FFQ data using PCA: ‘Snacks’ and ‘Fruit and Vegetables’. Though empirically derived dietary patterns are specific to the population of interest, similarities across studies are commonly seen, allowing for comparisons between populations. The ‘Snacks’ pattern and ‘Fruit and Vegetables’ patterns derived in this study resemble patterns in other studies with similar aged children across the Western world [22,23,24]. Whilst the naming of the patterns varies, the foods contributing the most to these patterns are comparable. For example, Oellingrath et al. [23] derived a ‘Varied Norwegian’ pattern in 924 children aged 9–10 years, which is similar to the ‘Fruit and Vegetables’ pattern derived in this study. Food groups that loaded positively in both of these patterns included fruit, vegetables, potatoes, cheese, yoghurt and brown bread. The results from this study show that boys have a higher ‘Snacks’ score compared to the girls. This finding is also similar to the Oellingrath et al. study [23] who found that boys had a higher score for their ‘Snacking’ pattern. It is not surprising that in both studies, boys had a more frequent consumption of treat and junk type foods that taste good due to their high fat and/or sugar content, as previous research has suggested that taste is a major influence in boys’ food preference and food choice [24]. Conversely, girls report to be more influenced by how healthy foods are than how they taste [25,26].

In the current study, parents’ diet quality was negatively associated with the children’s ‘Snacks’ pattern. This suggests that if parents have a poorer quality diet, their children’s overall diet consists of a more frequent consumption of less healthful foods. Though there are no other studies investigating the relationship between theoretically and empirically derived dietary patterns in parents and children, there is other evidence to suggest that diet quality are closely positively related between parents and children [7,13]. We found that adjustment for parental education had no real effect on any analyses but this is likely to be due to that fact that 71% of participating parents had post-secondary school level education.

Using an a priori dietary score to measure dietary quality in children is controversial, as some investigators believe further validation and longitudinal research is needed before these can be used in epidemiological studies [27]. The rationale behind this is that it is unknown which study designs and settings are most appropriate when utilising a priori scores to determine paediatric disease risk [27]. The use of PCA, and derivation of multiple also allows for investigation of the relationship between parental diet quality and different aspects of the child diet.

It is likely that the overall diets of 9–12 year-old children are associated with their parent’s diets due to the lack of autonomy children have at this age in regard to dietary choices. Children in this age range consume at least two-thirds of their meals at home and are provided with nearly all of their food by their parents [13]. The literature convincingly suggests that many individual foods consumed by children are influenced by their parent’s intakes [6,7,8,9]. At this age, parents are considered to be one of the strongest influences on children’s diets [28,29,30]. It is therefore interesting that in the current study only certain combinations of foods and drinks that loaded strongly in the patterns were significantly associated with parent diet quality (‘Snacks’ pattern), while others were not (‘Fruit and Vegetables’ pattern). It may be that associations are attenuated as some parental questionnaires were completed by the child’s male caregiver. The non-quantitative nature of the child FFQ may also explain these results. However, it may also be because the snack type foods are the ones that children may have more autonomy over, rather than those provided in main meals. 

It is important to remember that the cross-sectional nature of this study means that directions of relationships cannot be measured. However, if we could assume that the parental diet influences the diet of their child, then improving parent diet quality has the potential to positively change children’s diets. Data from the 2014–2015 New Zealand Health Survey shows that approximately 64% of adults consume three or more servings of vegetables daily and 57% consume two or more servings of fruit daily [31]. If more New Zealand parents can reach these targets, then the likelihood of their children also doing this may be increased. However, given the fact that significant relationships were only seen for the “Snacks” pattern, a focus on reducing consumption of less healthy foods by parents may be a more effective strategy. 

As children grow into adolescents, the association between parents’ and children’s diets may weaken due to increased autonomy and greater influences outside of the home, such as school, jobs and social activities [32]. Previous research showing that the odds ratio for healthy diet agreement between parents and children falling from 4.05 for 2–10 years old, to 1.55 for children and adolescents older than 10 years [9]. Such findings highlight how the influences of children’s diets change during the transition from childhood to adolescence, making this a key time to optimise food intake. Previous research shows that while dietary habits track from childhood into adolescence and adulthood [1], healthy eating habits also decline, particularly during the transition from childhood to adolescence [33]. Therefore, by providing a family environment that optimizes the development of healthy eating before adolescence, the effects of a decline in healthy eating during adolescents may be less marked. More recent research [34] has also shown that parental influence is also associated with healthier diet behaviours in adolescence. However, it may well be that this is due to maintenance or adaptation of already learned behaviours, rather than learning new behaviours. 

Some limitations of this study include the use of a FFQ to measure the children’s dietary intake, which only measures frequency, not amounts. In addition to this, the FFQ was relatively short with only 28 items, meaning the entire diet may not have been extensively covered. Despite this, the FFQ was found to be a valid tool for ranking participants according to food group intake [15] and was an appropriate method for this study, where multiple questionnaires were being administered. The DHQ was previously validated in almost 4000 adults so was suitable to be used in this study (unpublished data). Furthermore, although the sample of participants in PEDALS is representative of the Dunedin population it is not nationally representative, as there is a lower proportion of Māori and Pacific people in Dunedin, compared to the whole of New Zealand [33]. This is also reflected in the high proportion of those from higher decile schools and areas of low neighbourhood deprivation and the lower levels of overweight and obesity compared to national level data [35]. However, the schools who were not chosen to take part, or who declined participation, were not markedly different in decile from those who participated and the majority of schools not participating were those of higher deciles. Also, the sample is representative of the population of the South Island of New Zealand [35] and as the majority of New Zealanders are of ‘New Zealand European or Other’ ethnicity, the results of the current study are likely to be applicable to the majority of New Zealand children. 

This study has several strengths, in particular the use of dietary patterns, rather than selected food groups. This is advantageous as dietary patterns look at the diet as a whole. All PCA analyses require some potentially subjective decisions to be made, but this was minimised through the use of standardised methods to group foods and naming patterns in a similar way to previous studies. Sufficient children were recruited into the PEDALS Study to meet the requirements for PCA. At least ten participants per food group entered into PCA are required in order to obtain robust results [36] and the study sample more than met this. Lastly, all questionnaires used were previously tested and validated in similar populations to this sample.

## 5. Conclusions

The current study found an association between parental diet quality and selected children’s dietary patterns. Parents with a poor diet quality were more likely to have children that had a frequent consumption of ‘Snacks’. This highlights the need for further investigation into the relationship between parent and child dietary patterns, as poor childhood diet quality remains prevalent in large parts of the world.

## Figures and Tables

**Table 1:** Characteristics of child and parent PEDALS participants.

Characteristic	Total Children (*n* = 401)		Boys (*n* = 198)		Girls (*n* = 203)		Total Parents (*n* = 401)		Fathers (*n* = 66)		Mothers (*n* = 335)	
	n	(%)	n	(%)	n	(%)	n	(%)	n	(%)	n	(%)
Age (years) *	10.2	0.6	10.3	0.6	10.2	0.6	41.6	5.5	43.5	6.3	41.3	5.3
Ethnicity												
Māori	36	9	23	12	13	6	21	5	2	3	19	6
Pacific	11	3	4	2	7	4	4	1	0	0	4	1
NZEO	354	88	171	86	183	90	376	94	64	97	312	93
BMI ^†^												
Underweight/normal	292	73	144	73	148	73	201	50	20	30	181	54
Overweight	66	16	27	13	39	19	133	33	33	50	100	30
Obese	43	11	27	14	16	8	67	17	13	20	54	16
School Year												
Year 5	227	57	104	53	123	61			37	56	190	57
Year 6	174	43	94	47	80	39			29	44	145	43
School decile												
Low (1–3)	26	6	13	7	13	6			6	9	20	6
Medium (4–7)	147	37	67	34	80	39			28	42	119	36
High (8–10)	228	57	118	59	110	55			32	49	196	58
NZDep13 ^§^												
Low (1–3)	180	45	99	50	81	40			31	47	149	44
Medium (4–6)	151	38	66	33	85	42			18	27	133	40
High (7–10)	70	17	33	17	37	18			17	26	53	16
DQI score ^||^ (range from 0–60)							43	7	40	6	44	7

* Presented as mean and standard deviation; ^†^ World Health Organisation (WHO) criteria used to derive and allocate BMI categories; ^‡^ Ratings given to schools in New Zealand to determine government funding; deciles range from 1 (low) to 10 (high). The lower the decile, the more funding received; ^§^ The New Zealand Deprivation Index 2013; ^||^ Diet Quality Index.

**Table 2:** Factor loadings (orthogonal varimix rotation) of food items/groups in two identified dietary patterns in 9–11 year-old children in PEDALS.

Food Items/Group	Snacks	Fruit and Vegetables
Fruits	−0.10	0.33
Vegetables (including potato) *	−0.01	0.36
Trim milk (green) [including on cereals, milo, hot chocolate]	−0.03	0.31
Milk (blue) [including on cereals, milo, hot chocolate]	0.02	0.29
Cheese	0.00	0.33
Yoghurt	0.01	0.33
Ice cream	0.28	0.02
Processed meat (such as meat pies, sausage, sausage roll, salami, luncheon, bacon, ham)	0.12	0.25
Other meats (such as mince, beef, chicken)	0.12	0.27
Fish (including canned tuna or salmon, fish cakes, fish fingers, fish pie, battered fish)	0.18	0.14
Non-dairy drinks ^†^	0.31	0.02
Breakfast cereals	0.00	0.25
White bread	0.26	−0.05
Brown /Wholemeal bread	−0.12	0.32
Rice, rice based dishes	0.16	0.10
Pasta (such as spaghetti, macaroni), noodles	0.24	0.12
Salty snacks ^‡^	0.40	−0.05
Biscuits, cakes, muffins, doughnuts, fruit pies	0.35	−0.04
Lollies/sweets	0.33	−0.09
Sweet snacks ^§^	0.33	0.02
Spreads ^||^	0.29	0.02
Eigen value	5.36	2.60
Variance explained	25.5%	12.4%

* Vegetables: “vegetables” + “Potato (such as mashed, boiled)”; † Non-dairy drinks: “Fruit juice (such as Orange juice, Apple juice, Raro, Refresh, Keri, Twist, Ribena”; + “Diet fizzy drinks (such as Diet Coke, Pepsi Max, Sprite Zero and any other “light” or “sugar free” varieties)” + ”Fizzy drinks (such as Coke, Pepsi, Sprite, L&P, Fanta, Ginger Beer)”; ‡ Salty snacks: “Potato chips, potato snacks, corn chips” + “Hot chips, wedges, French fries”; § Sweet snacks: “Snack bars (such as muesli bar, fruit bar, rice bubble bar)” + “Chocolate, Chocolate bars”; || Spreads: “Tomato sauce, Ketchup” + “Peanut butter, Nutella” + “Jam, honey”.

**Table 3:** Associations between children dietary patterns and parent Diet Quality Index Scores.

	DQI Score					
	Unadjusted			Adjusted *		
	β	(95% CI)	*p*	β	(95% CI)	*p*
‘Snacks’	−0.04	−0.05, −0.02	<0.001	−0.03	−0.04, −0.02	<0.001
‘Fruit and Vegetables’	0.01	−0.002, 0.03	0.091	0.01	−0.001, 0.03	0.060

* Adjusted for parent age, sex and BMI, and child age, sex, ethnicity and BMI *z*-score, and level of deprivation (NZDep13).

## References

[1] Craigie A.M., Lake A.A., Kelly S.A., Adamson A.J., Mathers J.C. (2011). Tracking of obesity-relatedbehaviours from childhood to adulthood: A systematic review. Maturitas.

[2] Kaikkonen J.E., Mikkilä V., Raitakari O.T. (2014). Role of childhood food patterns on adult cardiovascular disease risk. Curr. Atheroscler. Rep..

[3] Patrick H., Nicklas T.A. (2005). A review of family and social determinants of children’s eating patterns and diet quality. J. Am. Coll. Nutr..

[4] Crockett S.J., Sims L.S. (1995). Environmental influences on children’s eating. J. Nutr. Educ..

[5] Wardle J., Cooke L. (2008). Genetic and environmental determinants of children’s food preferences. Br. J. Nutr..

[6] Pearson N., Biddle S.J.H., Gorely T. (2009). Family correlates of fruit and vegetable consumption in children and adolescents: A systematic review. Public Health Nutr..

[7] Robinson L.N., Rollo M.E., Watson J., Burrows T.L., Collins C.E. (2015). Relationships between dietary intakes of children and their parents: A cross-sectional, secondary analysis of families participating in the Family Diet Quality Study. J. Hum. Nutr. Diet..

[8] Wolnicka K., Taraszewska A.M., Jaczewska-Schuetz J., Jarosx M. (2015). Factors within the family environment such as parents’ dietary habits and fruit and vegetable availability have the greatest influence on fruit and vegetable consumption by Polish children. Public Health Nutr..

[9] Wang Y., Beydoun M.A., Li J., Liu Y., Moreno L.A. (2011). Do children and their parents eat a similar diet? Resemblance in child and parental dietary intake: Systematic review and meta-analysis. J. Epidemiol. Community Health.

[10] Michels K.B., Schulze M.B. (2005). Can dietary patterns help us detect diet–disease associations?. Nutr. Res. Rev..

[11] Newby P.K., Tucker K.L. (2004). Empirically derived eating patterns using factor or cluster analysis: A review. Nutr. Rev..

[12] Lazarou C., Newby P.K. (2011). Use of dietary indexes among children in developed countries. Adv. Nutr..

[13] Beydoun M., Wang Y. (2009). Parent-child dietary intake resemblance in the United States: Evidence from a large representative survey. Soc. Sci. Med..

[14] Robson S.M., Couch S.C., Peugh J.L., Glanz K., Zhou C., Sallis J.F., Saelens B.F. (2016). Parent diet quality and energy intake are related to child diet quality and energy intake. J. Acad. Nutr. Diet..

[15] Atkinson J., Salmond C., Crampton C. (2014). NZDep2013 Index of Deprivation User's Manual.

[16] Saeedi P., Skeaff S.A., Eiin Wong J., Skidmore P.M.L. (2016). Reproducibility and relative validity of a short food frequency questionnaire in 9–10 year-old children. Nutrients.

[17] A Focus on Nutrition: Key Findings of the 2008/09 New Zealand Adult Nutrition Survey. https://www.health.govt.nz/system/files/documents/publications/a-focus-on-nutrition-v2.pdf.

[18] de Onis M., Onyango A.W., Borghi E., Siyam A., Nishida C., Siekmann J. (2007). Development of a WHO growth reference for school-aged children and adolescents. Bull. World Health Organ..

[19] Osborne J., Costello A. (2009). Best practices in exploratory factor analysis: Four recommendations for getting the most from your analysis. Pan-Pacific Manag. Rev..

[20] Wong J.E., Skidmore P.M.L., Williams S.M., Parnell W.R. (2014). Healthy dietary habits score as an indicator of diet quality in New Zealand Adolescents. J. Nutr..

[21] A National Survey of Children and Young People’s Physical Activity and Dietary Behaviours in New Zealand: 2008/09. https://www.health.govt.nz/system/files/documents/publications/cyp-physical-activity-dietary-behaviours-08-09-keyfindgs.pdf.

[22] Aranceta J., Perez Rodrigo C., Ribas L., Serra-Majem L. (2003). Sociodemographic and lifestyle determinants of food patterns in Spanish children and adolescents: The enKid study. Eur. J. Clin. Nutr..

[23] Oellingrath I.M., Svendsen M.V., Brantsæter A.L. (2010). Eating patterns and overweight in 9- to 10-year-old children in Telemark County, Norway: A cross-sectional study. Eur. J. Clin. Nutr..

[24] Nu C.T., MacLeod P., Barthelemy J. (1996). Effects of age and gender on adolescents’ food habits and preferences. Food Qual. Prefer..

[25] Sweeting H.N. (2008). Gendered dimensions of obesity in childhood and adolescence. Nutr. J..

[26] Weible D. (2013). Gender-driven food choice: Explaining school milk consumption of boys and girls. J. Consum. Policy.

[27] Marshall S., Burrows T., Collins C.E. (2014). Systematic review of diet quality indices and their associations with health-related outcomes in children and adolescents. J. Hum. Nutr. Diet..

[28] Brug J., Tak N., te Velde S., Bere E., de Bourdeaudhuij I. (2008). Taste preferences, liking and other factors related to fruit and vegetable intakes among schoolchildren: Results from observational studies. Br. J. Nutr..

[29] Davison K.K., Birch L.L. (2001). Childhood overweight: A contextual model and recommendations for future research. Obes. Rev..

[30] Koui E., Jago R. (2008). Associations between self-reported fruit and vegetable consumption and home availability of fruit and vegetables among Greek primary-school children. Public Health Nutr..

[31] Ministry of Health (2015). Tier 1 Statistics 2014/15: New Zealand Health Survey.

[32] Videon T.M., Manning C.K. (2003). Influences on adolescent eating patterns: The importance of family meals. J. Adolesc. Health.

[33] Birch L., Savage J.S., Ventura A. (2007). Influences on the development of children’s eating behaviours: From infancy to adolescence. Can. J. Diet. Pract. Res.

[34] Draper C.E., Grobler L., Micklesfield L.K., Norris S.A. (2015). Impact of social norms and social support on diet, physical activity and sedentary behaviour of adolescents: A scoping review. Child Care Health Dev..

[35] 2013 Census QuickStats about Culture and Identity. http://www.stats.govt.nz/Census/2013-census/profile-and-summary-reports/quickstats-culture-identity.aspx.

[36] Floyd F., Wildaman K. (1995). Factor analysis in the development and refinement of clinical assessment instruments. Psychol. Assess..

